# Design and Development of a Diagnostic System for a Non-Intercepting Direct Measure of the SPIDER Ion Source Beamlet Current

**DOI:** 10.3390/s23136211

**Published:** 2023-07-07

**Authors:** Tommaso Patton, Alastair Shepherd, Basile Pouradier Duteil, Andrea Rigoni Garola, Matteo Brombin, Valeria Candeloro, Gabriele Manduchi, Mauro Pavei, Roberto Pasqualotto, Antonio Pimazzoni, Marco Siragusa, Gianluigi Serianni, Emanuele Sartori, Cesare Taliercio, Paolo Barbato, Vannino Cervaro, Raffaele Ghiraldelli, Bruno Laterza, Federico Rossetto

**Affiliations:** 1 Consorzio RFX, 35127 Padua, Italy; alastair.shepherd@igi.cnr.it (A.S.); basile.duteil@igi.cnr.it (B.P.D.); andrea.rigoni@igi.cnr.it (A.R.G.);; 2 Culham Centre for Fusion Energy, Culham Science Centre, United Kingdom Atomic Energy Authority, Abingdon, Oxfordshire OX14 3DB, UK; 3 Swiss Plasma Center (SPC), Ecole Polytechnique Fédérale de Lausanne (EPFL), 1015 Lausanne, Switzerland; 4 Institute for Plasma Science and Technologies of National Research Council (ISTP-CNR), 35127 Padua, Italy; 5 Centro Ricerche Fusione (CRF), University of Padua, 35127 Padua, Italy; 6 Department of Management and Engineering, University of Padua, 36100 Vicenza, Italy

**Keywords:** neutral beam injector, negative ions accelerator, beam fluctuations, SPIDER, ITER

## Abstract

Stable and uniform beams with low divergence are required in particle accelerators; therefore, beyond the accelerated current, measuring the beam current spatial uniformity and stability over time is necessary to assess the beam performance, since these parameters affect the perveance and thus the beam optics. For high-power beams operating with long pulses, it is convenient to directly measure these current parameters with a non-intercepting system due to the heat management requirement. Such a system needs to be capable of operating in a vacuum in the presence of strong electromagnetic fields and overvoltages, due to electrical breakdowns in the accelerator. Finally, the measure of the beam current needs to be efficiently integrated into a pulse file with the other relevant plant parameters to allow the data analyses required for beam optimization. This paper describes the development, design and commissioning of such a non-intercepting system, the so-called beamlet current monitor (BCM), aimed to directly measure the electric current of a particle beam. In particular, the layout of the system was adapted to the SPIDER experiment, the ion source (IS) prototype of the heating neutral beam injectors (HNB) for the ITER fusion reactor. The diagnostic is suitable to provide the electric current of five beamlets from DC up to 10 MHz.

## 1. Introduction

SPIDER [1] (Source for the Production of Ions of Deuterium Extracted from a Radio frequency plasma) is the ion source (IS) full-scale prototype of the ITER heating neutral beam (HNB) and has been operating at the Padova Neutral Beam Test Facility (NBTF) since June 2018. The main purpose of the SPIDER experiment is to optimize the ITER HNB IS, and it is currently the largest radiofrequency-driven negative ion source operating in the world.

The main beam diagnostics are beam emission spectroscopy, visible imaging and the diagnostic calorimeter STRIKE [2], and none of those systems can provide a direct measurement of the beamlet current. In the past, the beamlet current has always been indirectly estimated by STRIKE; which consists of 16 unidirectional carbon fiber–carbon matrix (CFC) composite tiles, placed downstream of the set of grids that accelerate the beam. The tiles are exposed to the beam, and their temperatures are recorded using two infrared cameras. The beamlet current is then estimated from the thermal footprint of each beamlet via calorimetry, thanks to the moderate broadening of the temperature profile guaranteed by the anisotropy of CFC. However, this measurement also takes into account the thermal effect of neutral particles generated inside the accelerator (i.e., H^0^ generated by stripping part of the negative ions) and passing through the grid apertures, and it has a low time resolution. For this reason, an electrical measurement was introduced to measure the current collected by each tile of STRIKE, which required a positive bias of the tiles to recollect the electrons emitted from the surface due to the impact of energetic particles from the beam. While this additional measurement provides useful information, it has some important drawbacks as only the average beamlet current of each tile can be extrapolated, losing the possibility of a spatial distribution of the beam on a scale smaller than the beamlet group. Moreover, since the tiles are positively biased, electrons generated between the last grid of the electrostatic accelerator and STRIKE, via the interaction of the beam with the background gas, are also collected from the electric measurement, leading to an overestimation of the beam current.

In this framework, the development of a new diagnostic system capable of precisely measuring the beamlet current with satisfactory time and spatial resolution is particularly advised and the purposes are several. Firstly, to directly measure the beamlet current to compare it to established SPIDER diagnostics. Secondly, for the possibility of assessing the beam uniformity, utilizing the beamlet separation. Finally, to investigate whether the RF field and a beating frequency in the plasma due to the differing oscillator frequencies affect the beamlet’s current, and thus optics [3]. For these reasons, a new beam diagnostic called beamlet current monitor (BCM) was designed, developed, installed, commissioned and tested in SPIDER.

## 2. SPIDER

The ITER HNBs are required to supply 16.5 MW power each, with beam particles (hydrogen or deuterium) electrostatically accelerated up to 1 MeV and with a divergence lower than 7 mrad [4]. The plasma generation in neutral beam ion sources is typically conducted either by filament arc discharge (e.g., the negative ion source for neutral beam injection at JT-60 [5]) or via inductive coupling, as is performed at IPP-Garching’s BUG [6] and ELISE [7] facilities. The plasma generation in SPIDER is based on the latter system, by upscaling the design of the RF-driven BUG and ELISE ion sources.

In SPIDER, the plasma is generated inside the IS by eight driver coils arranged in a 4 × 2 matrix; the driver coils of each row are connected in series and powered by a 200 kW self-excited push–pull RF generator. Each generator can oscillate within a 1 MHz ± 0.1 MHz range, and the self-oscillation frequency is imposed by the plasma parameters and by an adjustable variable capacitor C_v_ located at the output of each oscillator. For each oscillator, the value of C_v_ is adjusted to match the load impedance (transmission line, matching network, plasma equivalent impedance and stray parameters of the circuit), resulting in a variation of several kHz among the four generators since the load impedance is not identical for all the generators. Furthermore, the working frequency of each oscillator cannot assume any value (by adjusting C_v_) within the oscillator frequency range due to the frequency flip phenomenon [8] which consists of an uncontrolled jump of the oscillator frequency when operating close to the resonant frequency of the load. This phenomenon further reduces the possibility to obtain good matching of the load with the same oscillation frequency for all generators.

The negative ions are generated inside the IS by volume or by surface production mechanisms, with the latter using evaporated cesium in the source to decrease the work function of the H^−^ production surfaces. With Cs, the extracted current density increases by around a factor of 5–10 [9]. To optimize the H^−^ extraction, a transverse magnetic filter is generated inside the ion source to confine fast electrons in the upstream region of the IS (driver region), avoiding the H^−^ destruction by high-energy electrons in the proximity of the plasma grid (PG), which closes the plasma chamber. This magnetic field is generated by DC flowing vertically inside the PG and returning via a busbar system, which can be regulated from 0 A up to 5 kA.

The negative ions are then extracted from the IS and accelerated by a three-grid electrostatic accelerator composed of the PG, extraction grid (EG) and grounded grid (GG) which can be biased up to −108 kV, −96 kV and 0 V, respectively, with respect to the ground potential. Each grid presents 1280 apertures divided into 16 beamlet groups, formed by 16 rows with five beamlets each, arranged in a 4 × 4 matrix. Embedded magnets are also present in the EG to suppress the co-extracted electrons [1] by bending their trajectory without substantially affecting the trajectories of the negative ions due to their much larger Larmor radius. The ion deflection by the suppression magnets is adjusted at the GG via electrostatic or magnetic compensation; the former via the displacement of the GG holes and the latter by embedded magnets inside the GG. Finally, a ferromagnetic sheet is placed in contact with the downstream side of the GG to confine the magnetic field inside the accelerator.

Since the beginning of the SPIDER operation, electric arcs involving the back of the IS started to arise when the vessel pressure was above 40 mPa (corresponding to 0.1 Pa inside the IS for H_2_) under some experimental conditions due to large RF voltages [10]. Therefore, ahead of a major modification of the pumping system, a molybdenum mask of 0.25 mm thickness was fastened downstream of the PG (a set of pushers press the mask against the PG) to reduce the number of open apertures [11]. Initially, a mask with 80 open apertures was used, with a further reduction to 28 for Cs evaporation. This temporary solution provides a lower gas flow conductance between the IS and the vessel, allowing operation up to 0.45 Pa in the source with minimized RF discharge occurrence.

The presence of this newly available space between open grid apertures, a consequence of the use of the PG mask, was deemed a perfect opportunity to design and install a temporary diagnostic dedicated to the measurements of single beamlets.

## 3. BCM Concept Design

The design of the BCM is driven by the dual desire to measure both the DC and AC components of the accelerated beamlet current. The primary requirements, based on the experimental performance of SPIDER and the expected beam current with Cs evaporation, are summarized in Table 1.

Although the average current of a single beamlet was estimated to be about 3–5 mA via STRIKE calorimetry, the full scale of the BCM system was chosen by considering the SPIDER nominal beamlet current when operating with cesium close to the optimal parameters, since BCM is planned to operate in both the volume and surface production phases.

A resolution lower than 1 mA, i.e., the minimum current variation which can be resolved, was defined on the basis of the maximum non-uniformity allowed (10%) considering a beamlet current of 10 mA, corresponding to the expected accelerated current in the early phase of surface production with non-optimized source parameters. The sensitivity, i.e., the output voltage of the instrument over the input current, was chosen to be at least 5 mV/mA on the basis of the typical sensitivity of general-purpose oscilloscopes, also considering the installation of the diagnostic in a noisy environment such as SPIDER.

**Figure 1:**
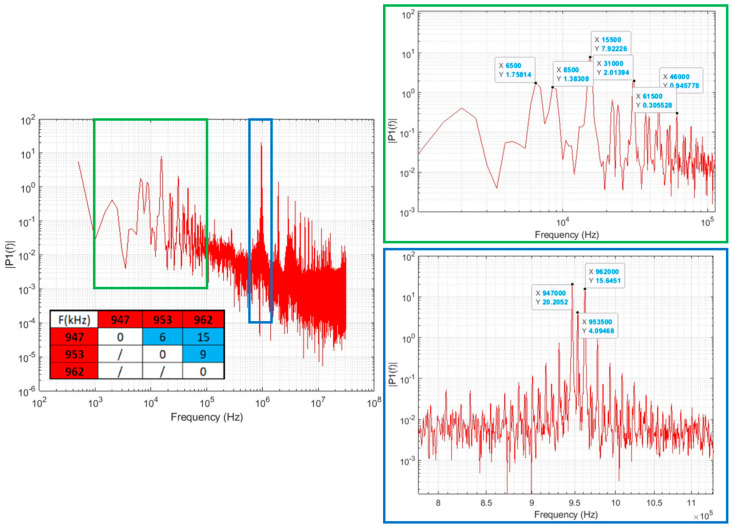
Langmuir triple probe signal, shot 8288, blip 3, volume operation with three out of four RF generators active. Generators working frequencies and related beating frequency are also shown in the red and light blue cells of the table (**left**). Zoom view around the beating frequencies (**top right**) and around the RF fundamental frequency (**bottom right**).

High-frequency fluctuations in the plasma, measured by a Langmuir triple probe [12], were identified at the fundamental frequency of the oscillators and their harmonics (Figure 1, highlighted blue). This observation is supported by the literature. High-frequency harmonics around the RF excitation field frequency may be present [13], and in particular, the ones up to three times the fundamental frequency seem to be the most relevant in terms of magnitude [14]. Peaks between 1 kHz and 100 kHz were also observed (Figure 1, highlighted green), with the lower frequency peaks compatible with the beatings of the power delivered to the plasma from each RF generator (blue cells of the table in Figure 1). For all the aforementioned reasons, a bandwidth from DC up to 10 MHz is a requirement of the BCM diagnostic.

Beyond the electrical requirements of the current transducer, the measurement system shall also be equipped with a repeller which is a positively biased copper electrode crossed by the beam and placed downstream of the GG. The purpose of the repeller is to prevent positive ions, generated between the GG and STRIKE due to beam–gas interactions, from entering backwards to the current transducers of the BCM under the effect of the stray electric field penetrating through the GG apertures in the downstream region [15]. Furthermore, the STRIKE positive bias also helps positive ions move backward towards the GG. The requirement in terms of repeller voltage was chosen as V_rep_ ≥ 100 V, which is a reasonable value for reducing the backstreaming ion current consistently since STRIKE is usually biased at 60 V.

**Figure 2:**
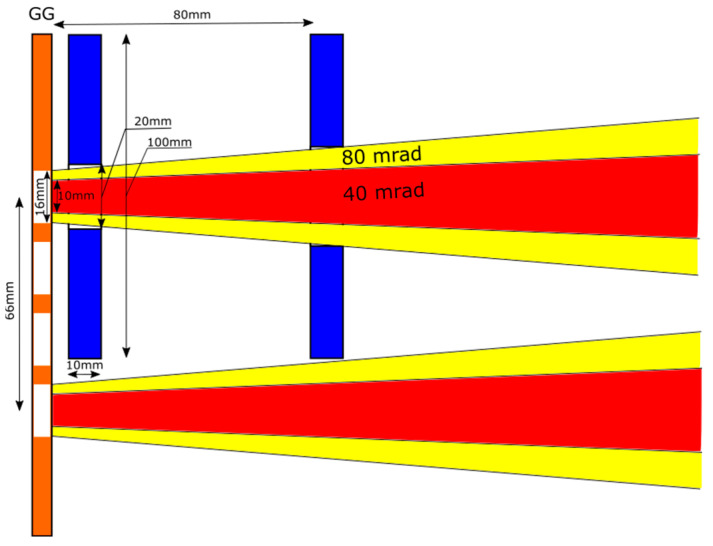
Vertical cross-section of 2 adjacent beamlets downstream of the GG (orange, apertures in white) with PG mask installed for the worst optics conditions (yellow cones) and for typical optics conditions (red cones); quotes are in mm. Example of minimum inner and outer diameters required for sensors (blue) depending on the distance from the GG.

Finally, the sensor size has also been assessed carefully, considering the available room, beam deflection, width and divergence downstream of the GG. This is a basic requirement since the interception of a steady-state high-energy beamlet (or part of it) would compromise not only the reliability of the BCM diagnostics but also the reliability of the whole SPIDER experiment. The sensor sizing was assessed in [2] by considering the worst conditions in terms of optics: 60 mrad divergence and 20 mrad horizontal deflection with a beamlet width at the exit of the GG equal to 16 mm, corresponding to the aperture diameter. In this conservative configuration, the minimum distance between the center of two adjacent apertures is 66 mm since the vertical pitch of the GG is 22 mm and a minimum of two apertures are masked between two beamlets, providing the maximum available space for the individual diagnostics (Figure 2). Therefore, the requirements concerning the sensor size were defined as clearance ≥ 20 mm and external diameter ≤ 100 mm depending on the distance from the GG (keeping below 200 mm).

## 4. Sensor R&D

### 4.1. AC Sensors

Two off-the-shelf current transformers (CTs) were chosen based on the high-frequency requirements, the BERGOZ ACCT-055 10 mA full scale (3 Hz–1 MHz) [16] and the Magnelab CT-F5.0_BNC (4.8 kHz–400 MHz) [17]. As an alternative to commercial CTs, a custom passive CT was designed and built in-house, based on the literature [18,19,20].

**Figure 3:**
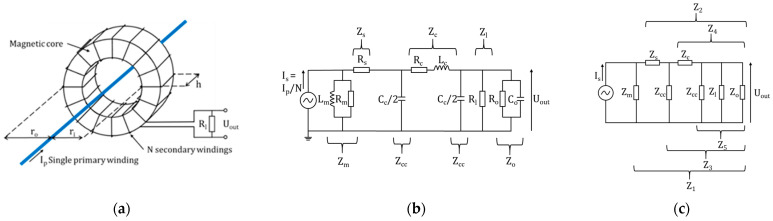
Current transformer: (**a**) diagram; (**b**) equivalent circuit; (**c**) equivalent circuit with grouped impedances for calculating the transfer function.

The current transformer (Figure 3a) consists of a magnetic core that surrounds the H^−^ beamlet. The secondary winding is wound around the core for N turns and terminated by a parallel load resistance $R_{l}$. The secondary winding has a magnetizing inductance that, along with the core losses, can be expressed as a parallel inductance $L_{m}$ and resistance $R_{m}$, with series resistance $R_{s}$, in the CT equivalent circuit (Figure 3b). To measure the transfer function, the sensor was connected to an HP 4194A Network Analyzer, with input impedance ($R_{0}$ parallel to $C_{0}$), via an RG58 coaxial cable with series inductance $L_{m}$ and resistance $R_{m}$ and parallel capacitance $C_{c}$, modeled as a π-junction.

The current through the secondary winding of the CT $I_{s}$ is the current of the primary winding $I_{p}$, in this case, the beamlet current, divided by the number of turns:

(1) $$I_{s}=\frac{I_{p}}{N}$$

For a simplified ideal current transformer, the gain in the mid-frequency range is:

(2) $$K=\frac{R_{l}}{N}$$

with the gain K dependent on the load resistance and the number of turns. A load resistance of 50 Ω and 10 turns were chosen to give the required 5 V/A gain. The low-frequency cutoff (−3 dB) $f_{low}$ of a CT:

(3) $$f_{low}=R_{l}/2\pi L_{m}$$

is dependent on $L_{m}$, since $R_{l}$ is chosen to set the gain. The impedance $L_{m}$ and losses $R_{m}$ of a magnetic core are dependent on the frequency, core permeability and geometry [21]. With the geometry limited by the available space around the beamlets, a high permeability material, nanocrystalline VITROPERM 500F [22,23], was chosen due to its high relative permeability $\mu_{r}=50,000$.

The CT was modeled in both MATLAB and PSIM, with the impedances in the MATLAB model grouped as in Figure 3c. By treating the circuit as a voltage divider, the transfer function can be calculated (see Appendix A):

(4) $$K=\frac{1}{N}.\frac{Z_{1}Z_{3}Z_{5}}{Z_{2}Z_{4}}$$

Using the MATLAB and PSIM models, a CT was designed to meet the gain, frequency and space requirements, with the parameters given in Table 2.

**Figure 4:**
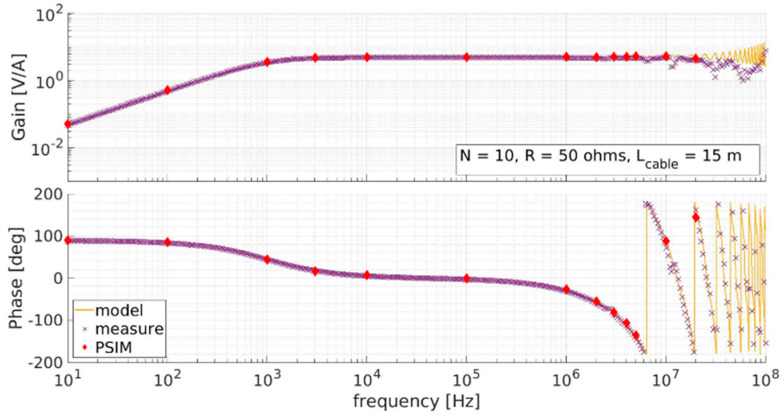
Measured CT response (purple) compared to MATLAB model (yellow) and PSIM model with 50 π junctions.

A CT prototype was constructed including a 15 m RG58 coaxial cable (the length required for the installation in SPIDER) connecting the CT to the 50 Ω load resistor. Figure 4 shows the measured response (purple crosses), which compares favorably with the MATLAB (yellow line) and PSIM (red points) models. At high frequencies, the gain and phase exhibit resonances, with the gain oscillations damped below 20 MHz. These resonances are due to the transmission line, which at 15 m was modeled as 50 junctions, giving a closer match to the measured frequency response.

### 4.2. DC Sensors

**Figure 5:**
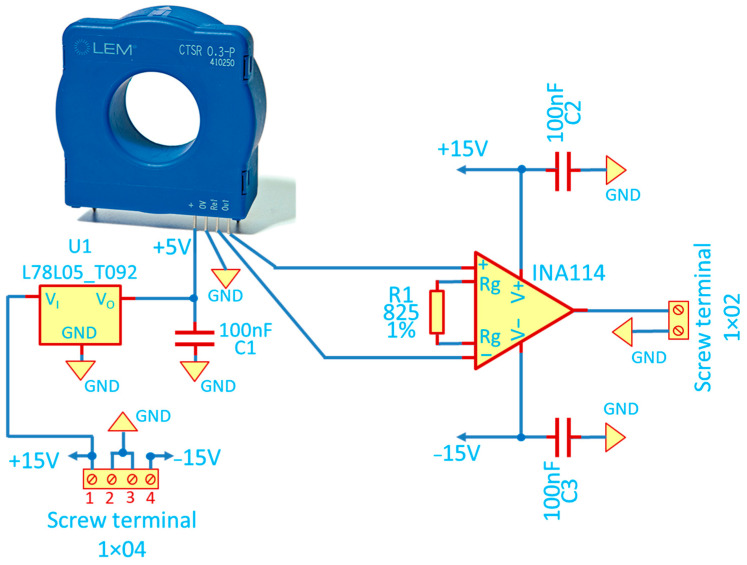
Schematic of the DC sensor with its signal conditioning circuit.

The sensor chosen to fulfill the DC and low-frequency measurements is the LEM CTSR 0.3p [24], a closed-loop fluxgate [25] with a sensitivity of 4 mV/mA. While the sensor fulfills most of the design requirements, this sensitivity is insufficient since the noise picked up on the cables from the sensor to the acquisition system may easily exceed this value. Additionally, the sensor has a “natural” offset of 2.5 ± 0.005 V, which must be removed to utilize the full range of the measuring oscilloscope, since it is not convenient to read a small quantity (e.g., few mV) when the full scale of the acquisition system ADC is higher than three orders of magnitude (e.g., FS > 2500 mV). A custom conditioning circuit was designed both to cancel out the offset (Figure 5) and to amplify the output signal to obtain a sensitivity of the order of 250 mV/mA. The circuit’s central component is the INA 114 instrumentation amplifier from Texas Instruments. Provided with an input signal $V_{in}$, a reference signal $V_{ref}$ (2.5 V in our case, taken directly from the LEN internal reference) and an interchangeable resistor $R_{g}$, the amplifier produces a signal $V_{out}$ given by:

(5) $$V_{out}=\left(V_{in}-V_{ref}\right)\left(1+\frac{50k\Omega }{R_{g}}\right)$$

For example, a resistor of 813 Ω (chosen value) provides a multiplication factor of 62.5 and, taking into account the sensitivity of the sensor, a final gain of 250 mV/mA.

### 4.3. Mounting Structure

**Figure 6:**
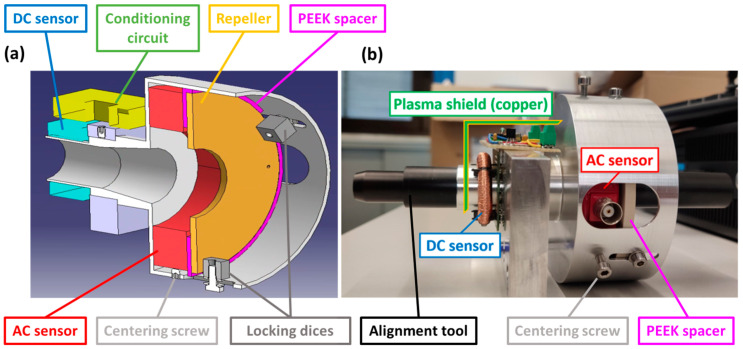
Mounting structure with AC and DC sensors: (**a**) model (**b**); assembled sensor.

Each sensor group consists of a custom mounting structure containing the DC sensor, its conditioning circuit (protected from the plasma by a copper shield), the AC sensor and the repeller. The repeller is insulated from the grounded support structure by a PEEK spacer, and the structure is equipped with three PEEK locking dices and three centering screws to ensure a robust and adjustable ensemble (Figure 6). Each group is fixed to the PG mask pushers support structure downstream of the grounded grid, and an alignment tool was used to properly align the sensors with the beamlet aperture.

### 4.4. Vacuum Testing

Preliminary tests to assess the compatibility of the instruments for the installation inside SPIDER were carried out with a twofold purpose. Firstly, to evaluate the outgassing as a function of the temperature and identify the nature of the contaminants (e.g., halogens must be absolutely avoided, since they react vigorously with cesium to produce salts). Secondly, to check the functionality of the DC sensor in HV, since active electronics are present and therefore local overheating, due to self-heating, may occur, leading to a change in the transfer function.

**Figure 7:**
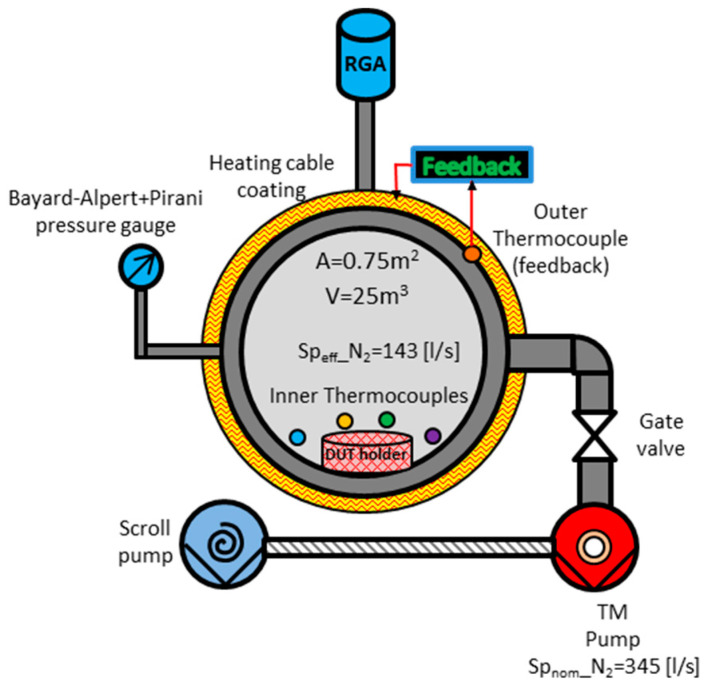
Scheme of the experimental setup adopted for the characterization of the instruments in a vacuum.

A dedicated test stand composed of a small stainless steel vacuum chamber (V ≈ 25.l, A = 0.75 m^2^), a turbomolecular pump (Leybold Turbovac TMP 361, Sp_nom__N2 = 345.l/s), a full-range pressure gauge (Leybold ITR90) combining Bayard-Alpert and Pirani sensors and a mass spectrometer (Inficon Transpector 2) were used. The wall of the chamber was covered by a heating cable wrapped around the outer side of the bake-out system, and, finally, some thermocouples were placed both on the outer side of the wall and inside the chamber; a sketch of the experimental setup is shown in Figure 7.

**Figure 8:**
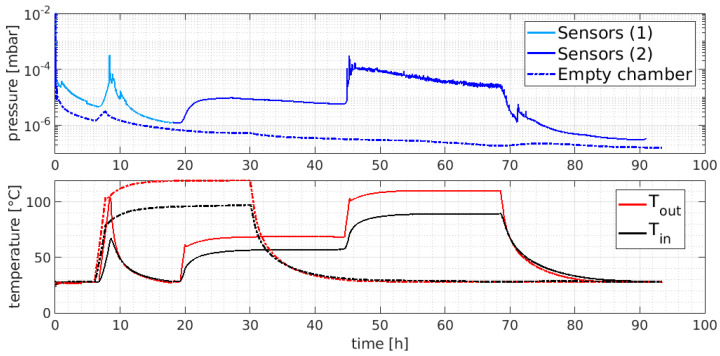
Pump-down curve of Magnelab CT-F5 (solid line) compared to that of the empty chamber (dashed line). Chamber temperature is shown in the bottom plot: red = outer thermocouple, black = inner thermocouple.

Before testing the instruments, the vacuum chamber was pumped down, and after 6 h, it was baked at 95 °C for 24 h, and then pumped at room temperature for up to 90 h until a base pressure of 2 × 10^−7^ mbar was reached. The bake-out temperature was chosen on the basis of the maximum rated temperature of the instruments to be tested so that the same cycle in principle could be applied to each sensor. The pump-down curve of the empty vacuum chamber is shown in Figure 8 (dashed line) where the typical t^−1^ dependency of the pressure due to the outgassing flux reduction over time is visible. The bake-out system, after an increase in pressure due to an increase in the desorbed gas flux stimulated by the temperature increase, allowed the chamber to approach its base pressure.

#### 4.4.1. AC Sensor Outgassing Tests

**Figure 9:**
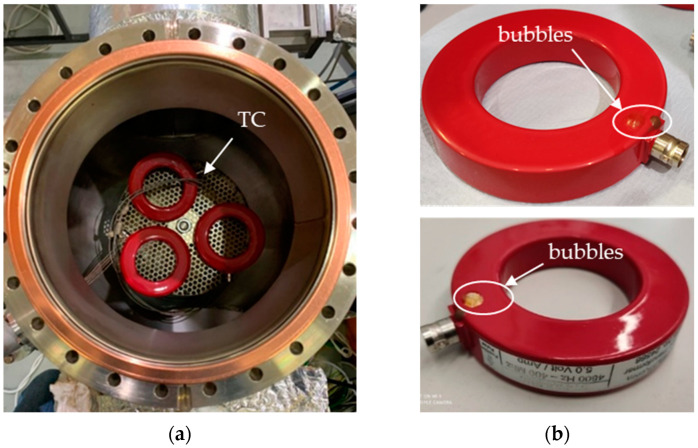
Magnelab CT: (**a**) in a vacuum chamber; (**b**) sample n° 3 (M3) after bake-out at 90 °C.

The off-the-shelf AC sensors (Magnelab CT-F5), being completely passive, were tested to assess the outgassing rate as a function of the temperature and their capability to stay in a vacuum (e.g., without explosions due to trapped air at room pressure inside it). Additionally, this test enabled us to degas the sensors to reduce the contamination of the SPIDER vacuum as much as possible since no information about their vacuum behavior was provided by the manufacturer. The pump-down curve of the Magnelab CT-F5s compared to that of the empty chamber is shown in Figure 8. All three samples foreseen for the diagnostics implementation in SPIDER were inserted into the chamber after a cleaning phase using acetone; the inner thermocouples were placed between the sample holder and the instruments as shown in Figure 9a.

The experiment involved long periods with sustained external heating, and it was possible to deduce that the sensors would behave normally below 45 °C, as can be seen by the fact that the oscillation in the pressure signal (supposedly due to boiling) disappeared and the pressure approached the 10^−7^ mbar decade after a fast drop.

**Figure 10:**
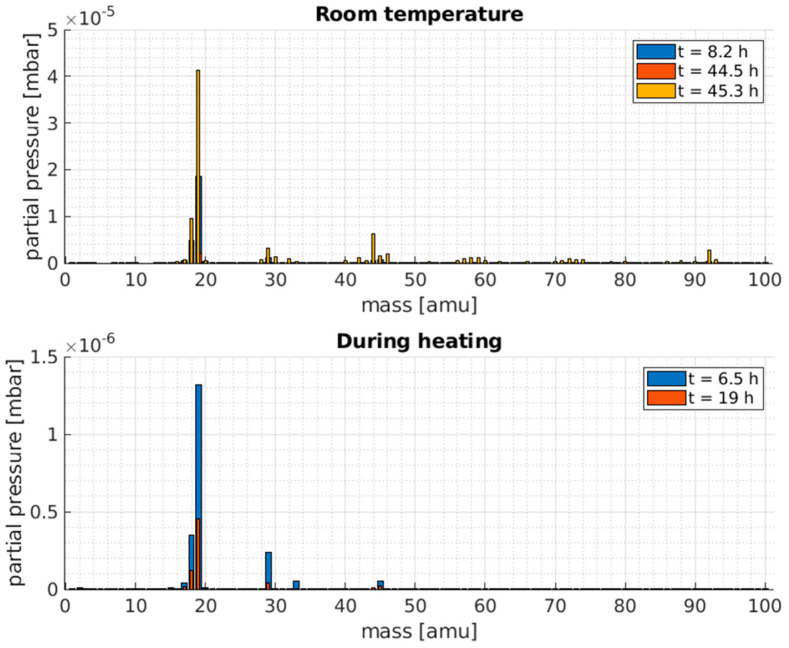
Residual gas analysis of Magnelab CT-F5: Partial pressure of all the species at different times during the bake-out experiment. The times at which the measurements were made are indicated on the labels.

The residual gas analysis (RGA) during the pump-down curve is shown in Figure 10 where the main contaminants appeared to be water vapor (mass 17 and 18) and nitrogen (mass 28) both for the room temperature case and for the hot sensor case. Nevertheless, for this latter, other, heavier contaminants appeared, in particular, mass 44 (C0_2_) and mass 92 (expected to be toluene, commonly used for fast-drying paints).

On the basis of this test, an additional requirement in terms of the range of working temperatures in the vacuum inside SPIDER was defined as t < 45 °C; for this purpose, a thermal analysis was also carried out and is reported in Section 4.4.3.

After the test, the sensors were removed from the vacuum chamber and visually inspected: numerous microbubbles were observed on the painted surface, and a big bubble on sample n° 3 (M3) in the proximity of the BNC connector was also present, as shown in Figure 9b. The transfer functions of all three samples were measured with a spectrum analyzer (HP 4194A) to check if the nominal one was preserved after the test in UHV. This was confirmed for sensors M1 and M2, while the M3 sensor reported a slightly higher value for its low cutoff frequency.

The custom current transformer based on the VITROPERM 500F material was also tested in a high vacuum. The nanocrystal material is made of a very thin ferrite tape (14 µm to 20 µm) which is very brittle and is therefore provided already coated with epoxy resin or enclosed in a high-quality (UL94-V0, Class F) sealed plastic casing. The plastic casing type was chosen since epoxy resin (Class A) was expected to be less stable with the temperature; nevertheless, the sealed plastic case was not vacuum-tight, and, therefore, two small holes were carefully made in the casing to avoid trapped volumes (interspace between the core and the case) and therefore virtual leaks when exposed to a vacuum. The baseline pressure obtained during the pump-down test was 2 × 10^−6^ mbar, which was considered an acceptable value. The fact that this value is slightly higher than the off-the-shelf sensors might be due to the much greater overall surface of the tape exposed to the vacuum (>1 m^2^), considering the dimensions of the core and the tape thickness, as well as the fact that no bake-out was applied during this test. Unfortunately, the results when external heating was applied are not reliable since the pressure gauge was not working properly, and they have been excluded.

However, the absence of reliable outgassing data at high temperatures was not considered limiting for the installation, since from thermal simulations (already carried out on the basis of the results of Magnelab CT), the expected working temperature should not exceed 35 °C.

#### 4.4.2. DC Sensor Vacuum Tests

**Figure 11:**
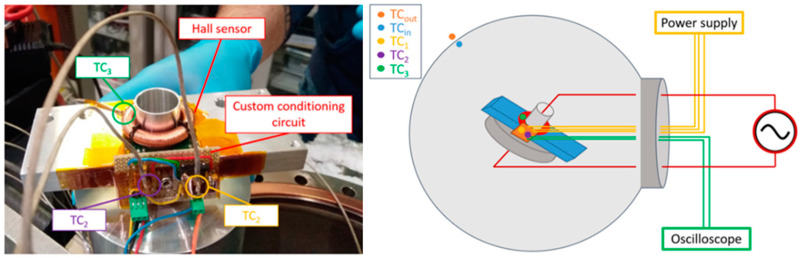
DC sensor experimental setup for the tests in a vacuum.

After initial benchmarking tests, the vacuum and temperature responses of the LEM sensor (together with the custom signal conditioning circuit) were tested. After removing its plastic casing to avoid trapped volumes, one of the DC sensors and its custom circuit were fastened to the mounting structure used for the installation in SPIDER, which also acts as a heat sink. Thermocouples were fixed to the critical components of the sensor, as well as on the vacuum chamber walls both on the inner and outer sides as shown in Figure 11.

**Figure 12:**
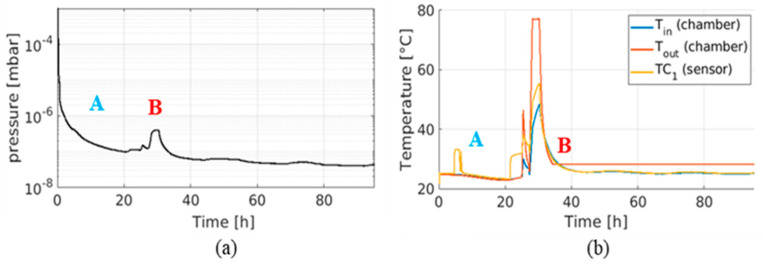
(**a**) Pump-down curves of DC sensor at room temperature and with external heating, (**b**) chamber temperature during the experiment (orange: outer thermocouple, blue: inner thermocouple, yellow: sensor thermocouple). Points A and B correspond to self-heating and external-heating phases, respectively.

The pump-down curve is shown in Figure 12a, at room temperature (point A) to assess the outgassing rate and the thermal management when operating in a high vacuum, where the temperature increase in the sensor is only due to self-heating, as well as with external heating (point B) to check the sensor transfer function when subjected to external heating. The sensor outgassing rate was relatively low since the pressure reached values below 1 × 10^−7^ mbar. In addition, the identified solution to manage the heat dissipation of the sensor was found to be satisfactory also during the external heating (50 °C chamber temperature and self-heating of the instrument).

The custom circuit designed to remove the sensor’s “natural” offset before amplification is not perfect, as V_in_–V_ref_ with no external current is not precisely zero. An offset of approximately 10 mV remains, rising to several volts after amplification. In bench testing, the offset has proved to be unstable, changing each time the sensor is powered on/off but then remaining constant during operation, even with external heating up to 60 °C. Therefore, the behavior of the offset in vacuum was assessed both at room temperature and with external heating applied.

**Figure 13:**
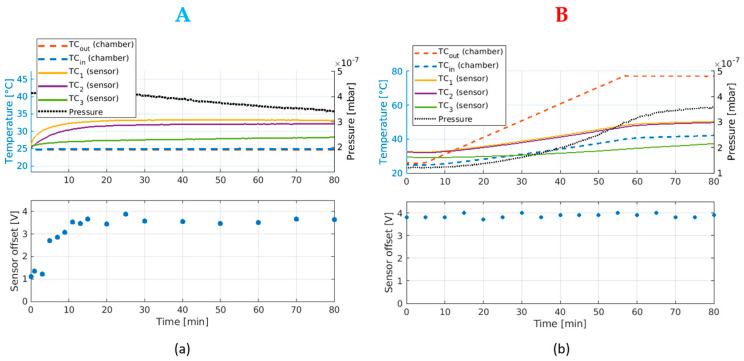
(**a**) DC sensor offset in vacuum at startup, with the vacuum chamber temperature kept constant at room temperature (point A of Figure 12), and (**b**) DC sensor offset in a vacuum while raising the ambient temperature (point B of Figure 12).

Figure 13a shows the evolution of the offset in the 80 min following the switching on of the sensor. In the first 10 min, the offset rises steadily until it reaches 3.5–4 V. It then remains at this level for the following 70 min. This behavior correlates to the rise in temperature of the sensor’s components only due to the self-heating produced by the power dissipated by the electronics.

The sensor is expected to reach higher temperatures in SPIDER when exposed to the ion beam. The same test was therefore performed by heating the vacuum chamber up to temperatures around 70–80 °C (Figure 13b) after reaching the thermal equilibrium due to the self-heating process only. The sensor’s temperature is sensitive to the raising temperature of its surroundings and reaches a maximum temperature of 50 °C, compared to 33–34 °C, when no external heating was applied to the chamber. However, this had no effect on the sensor’s offset, which remained in the order of 3.8–4 V. Any variation in the offset of an individual sensor is thus attributed to the initial rise in temperature after being switched on, stabilizing once a steady temperature is reached. This behavior is mandatory to have a reliable measurement, as the external heating due to the beam presence should not affect the offset and the measurement.

**Figure 14:**
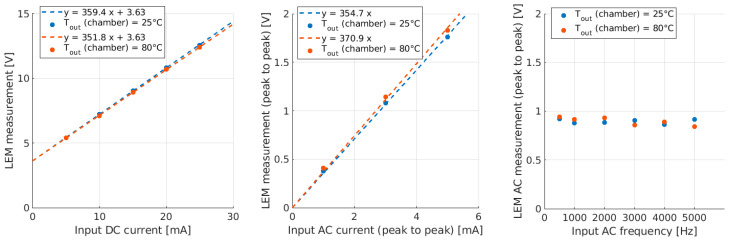
DC sensor response to flowing current at Tout = 25 °C and Tout = 80 °C.

Additional tests on the sensor’s response to a current in a vacuum, at 25 °C and 80 °C, were carried out (Figure 14). Three cases were investigated, the DC response (left), the AC response for signals at different amplitudes but at a fixed frequency of 1 kHz (center) and the AC response for signals at different frequencies but with a fixed amplitude of 5 mA peak-to-peak (right). In all three cases, the sensor’s performance seemed to change very little from one temperature to another. The sensor’s gain in these experiments changed by a factor of 5% at most, which is comparable to the measurement error and not considered problematic; thus, good linearity is achieved when the sensor works in a vacuum in a range of temperatures compatible with that expected in SPIDER.

#### 4.4.3. Thermal Simulations

Thermal simulations were carried out using Ansys to assess the temperature of the sensors during SPIDER operation, in particular, driven by the temperature limitation of the Magnelab sensor when operating in a vacuum due to the large and uncontrolled outgassing rate when the temperature of the sensor exceeded 45 °C (see Section 4.4.1).

The potentially most critical issue is represented by the fact that the PG mask can reach a relatively high temperature due to the input power given by the plasma; therefore, the PG mask temperature could reach an equilibrium value up to 300 °C, where the supplied power is mainly dissipated by radiative thermal transfer [11]. Therefore, the radiated power might also reach the BCM sensors since many direct lines of sight between the mask and the sensors are allowed by the EG and GG apertures.

**Figure 15:**
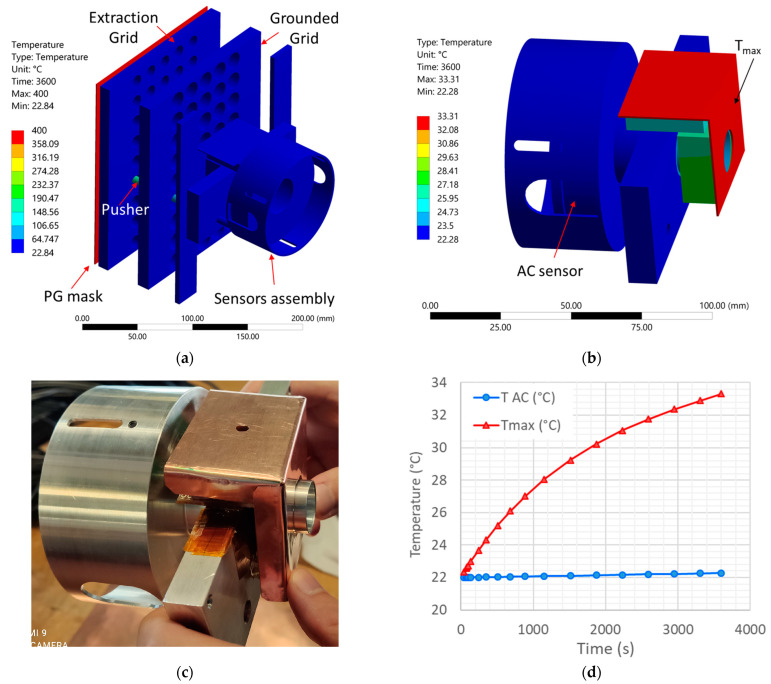
Transient thermal simulation of the BCM sensors assembly: (**a**) temperature at 3600 s in the whole simulation domain, (**b**) zoom view on the sensors assembly, (**c**) picture of the sensors assembly, (**d**) temperature on the hottest point (red curve) and on the AC sensor (blue curve).

The thermal simulation was carried out (Figure 15) considering only radiative heat transfer (conservative hypothesis), assuming the following boundary conditions:

- PG mask temperature set to 400 °C with emissivity equal to 1, representing the worst-case scenario (black body emitter, including a safety margin in the PG temperature);
- EG temperature set to 30 °C since it is actively cooled, and the emissivity of EG and GG set equal to 0.2 (slightly oxidized copper, the grids were exposed to air and humidity in the past [26]);
- Emissivity of pushers equal to 0.85 (pyrex glass [27]);
- Emissivity of the DC sensor and conditioning circuit equal to 0.75 (fiberglass of the electric circuit board [28]);
- Emissivity of plasma shield equal to 0.2 (slightly oxidized copper, the plate was exposed to air and humidity in the past [26]);
- Emissivity of sensors support structure equal to 0.1 (aluminum [27]);
- Emissivity of AC sensor equal to 0.03 (polished copper, the sensor surface was covered by copper tape to prevent plasma etching and to reduce the absorbed power by radiation [26]);
- Constant ambient temperature equal to 22 °C representing the vacuum vessel’s inner surface which is at room temperature.

The simulation time was limited to 3600 s, which is the maximum pulse duration foreseen for SPIDER operation; however, from Figure 15d, it can be seen that values are well within the safety limits. The maximum temperature reached on the instruments due to the radiated power from the PG mask is about 33 °C, on the copper plasma shield upstream of the DC sensor (red line in Figure 15d), whereas the temperature on the AC sensor is almost stable around the room temperature due to the shielding effect of the sensor’s mounting structure. In general, the radiated power reaching the sensors is relatively low due to the shadowing of the EG and GG grids; therefore, the installation of all the aforementioned sensors was considered reliable, considering the issues correlated to heat management.

## 5. Overview of the System

The BCM system is divided into the following main sub-systems: the sensors and the repeller disk (installed inside the vacuum vessel), cabling, the feedthroughs and surge arresters (installed on the vacuum/air feedthroughs), the data acquisition system, the power supply system and, finally, the CODAS database [29].

### 5.1. Sensor Installation

**Figure 16:**
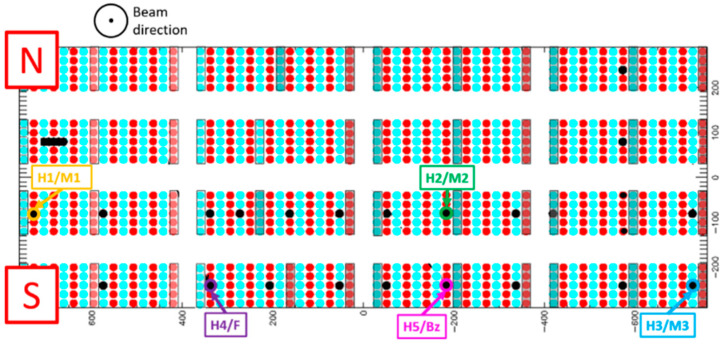
Positions of the five sets of sensors during the cesium campaign.

**Figure 17:**
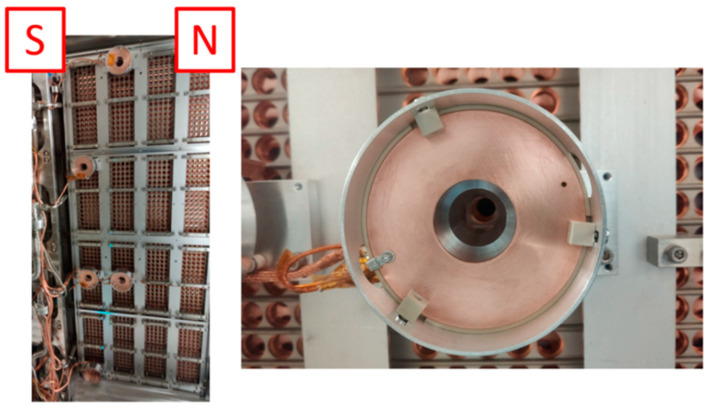
Installation in SPIDER (north and south are indicated to keep track of the orientation of the picture) showing the sensors fixed to the PG mask pusher structure.

The position of the five beamlets chosen for the BCM measurements during SPIDER’s first Cs campaign is shown in Figure 16. The beamlets are labeled according to the sensors making the group: the DC/low-frequency sensors (labeled H1 to H5) followed by the AC sensors (either Magnelab CT-F5s labeled M1 to M3, custom current transformer labeled F, or Bergoz ACCT 055 labeled Bz). North and south are indicated in the figure to better compare the positions of the sensor with the pictures taken inside the source during the installation (Figure 17).

The sensor’s mounting structure is metallically connected to the GG, which, during the accelerator breakdowns, can oscillate between several kV values with respect to the ground potential due to the stray inductance of the related ground connections.

Therefore, a reliable design was implemented to mitigate the risk of failure of the BCM system, which could also compromise the reliability of the whole SPIDER experiment (surge propagation to other subsystems and vacuum leak due to the rupture of the vacuum feedthroughs).

**Figure 18:**
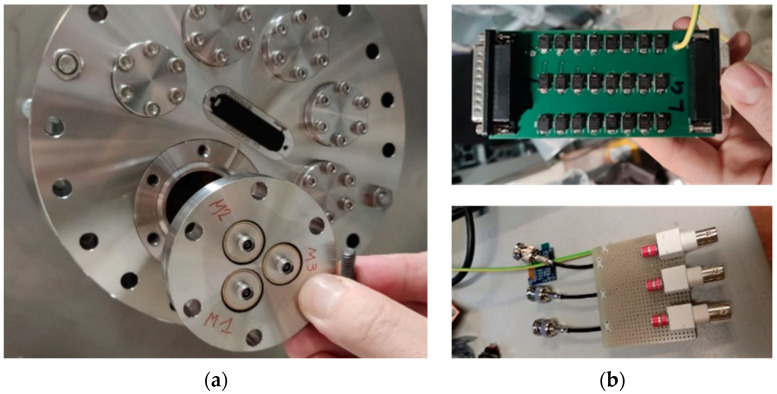
Pictures of: (**a**) the BCM feedthroughs; and (**b**) the related surge arresters: TVS PCB (**top figure**) and GDT board (**bottom figure**).

To avoid any additional paths to ground for the breakdown current through the BCM system itself, the instruments were insulated from the mounting structure by Kapton pads, and the sensor’s power supply as well as the data acquisition system were insulated from the ground. Finally, surge arresters were adopted to protect the feedthroughs (Figure 18).

### 5.2. Feedthroughs and Surge Arresters

The DC sensors, the Bergoz, the Custom CT, and the repeller cables were connected to a D-Subminiature feedthrough (900 V DC voltage), which was protected against overvoltage by a dedicated PCB, hosting a set of transient voltage suppressors (TVS). The selected transient voltage suppressors (TVS) were connected between each pin and the vacuum vessel (Figure 18b top).

A different choice was made for the Magnelab AC sensors since a 50 Ω matched line is required to exploit the maximum bandwidth of the instruments. Therefore, they were connected to 50 Ω SMA coaxial feedthroughs floating shield type (1 kV DC voltage) (Figure 18a). For these feedthroughs, three electrode GDTs were chosen (instead of TVS) as voltage suppressors due to the much lower stray capacitance (1.5 pF with respect to 300 pF) to limit, as much as possible, the effect of the surge arrester in the high-frequency range (Figure 18b bottom). The DC spark-over voltage is 350 V, whereas the impulse spark-over voltage is <900 V for slew rates of 1 kV/µs. The main drawbacks of this type of surge arrester with respect to TVS are the strong dependence of the pulse breakdown voltage to the slew rate, the lower number of operations within the service life and after arc ignition, they remain in the conductive state until the applied voltage is enough to sustain the arc current (crowbar behavior) [30]. This type of GDT, like the TVS, becomes a “virtual short” at the end of its life, always providing protection to the devices connected in parallel; therefore, the protection of the system is always assured.

### 5.3. Data Acquisition and Power Supply System

The data acquisition system was designed to be as modular as possible and with insulated channels so that any damage to a part of the system would not compromise the whole data acquisition system.

A relatively cheap solution that also allows the components to be easily replaced was identified in the STEMlab 125-14 Red Pitaya boards (RPs). Those boards have two analog inputs (±1 V or ±20 V, selectable), an input bandwidth at −3 dB of 60 MHz, a maximum sampling rate of 125 Msps and a 14-bit ADC. These boards can be remotely controlled and can be interfaced with the network or via WiFi or 1 Gbit Ethernet protocol.

**Figure 19:**
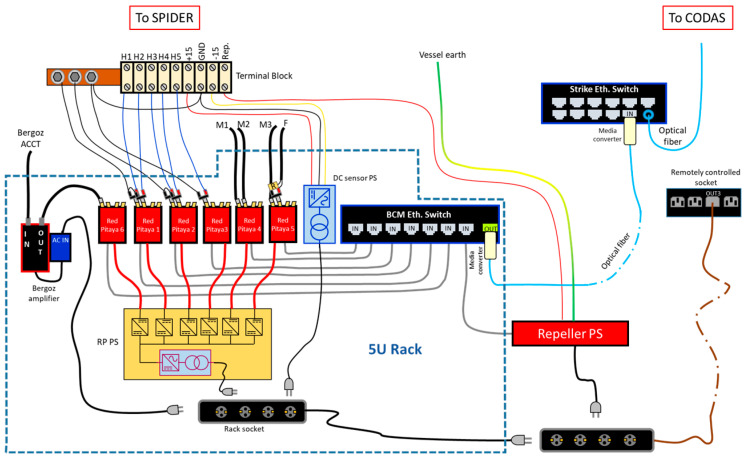
Overview of the BCM power supply and data acquisition system.

For the BCM system, six RPs were implemented to acquire the 10 sensors; the data communication for setting the parameters of the ADC and reading the acquired data is based on Ethernet (Figure 19).

The six RPs were powered by a set of six DC/DC converters (24 V/5 V) capable of assuring 2 kV DC insulation both among the devices and to the ground. The primary of the DC/DC converters was fed by a 230 V/24 V DC power supply rated 4 kV AC to withstand voltage, also providing an additional insulation barrier to the ground. Finally, this power supply was connected to the electric grid available inside the SPIDER bunker via a remotely controlled socket, which can switch the whole BCM system on and off.

Additionally, the data transmission had to assure proper insulation for the RPs and to the ground; therefore, a network switch with insulated ports was installed; this type of Ethernet port includes isolation transformers with a minimum isolation rating of 1500 VRMS (2.1 kV peak) as required by the IEEE 802.3 standard for Ethernet interfaces.

**Figure 20:**
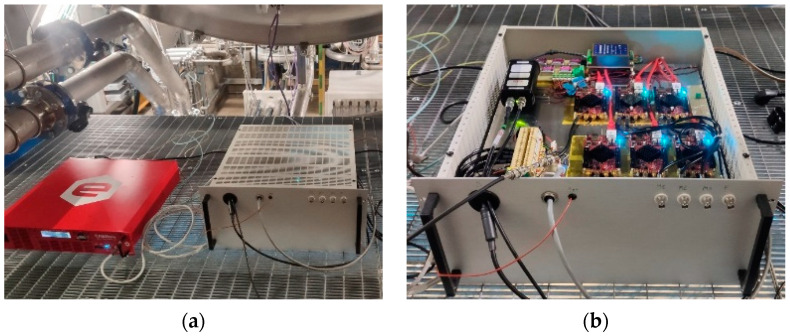
Picture of (**a**) the BCM power supply and (**b**) the data acquisition system.

The other power supplies are the DC sensor’s power supply, consisting of an AC/DC 230 V/±15 V power supply developed in RFX, and the Bergoz ACCT power supply AC/DC 230 V/±15 V rated at 4 kV RMS I/O to withstand voltage feeding the ACCT amplifier. All of the aforementioned components were enclosed in a 5U rack and placed inside the SPIDER bunker about 4 m away from the VV. Finally, the repeller’s power supply, a bipolar DC (−100 V < V < 100 V) power supply, was installed close to the BCM rack, and it was also connected to the Ethernet switch to allow it to be controlled remotely. One pole was connected to the VV and the other to the repeller disks so that only the plasma closed the circuit. The repeller power supply is shown in Figure 20a, along with the BCM rack, which houses the acquisition system in Figure 20b.

### 5.4. CODAS

The internal organization of the RedPitaya acquisition system exploits the recent system of chip (SoC) solution proposed by Xilinx, which embeds, in the same chip, a programmable logic (PL) built on a state-of-the-art 28 nm high-k metal gate (HKMG) technology FPGA, and a dual-core ARM Cortex-A9 MPCore processing system (PS). In particular, the RedPitaya mounts the Zynq 7010 system that provides an Artix-7 middle-range device. The key factor that characterizes this family of chips is not actually the performance of the components themselves but the high bandwidth that the FPGA is capable of sustaining when communicating with the other internal SoC components, i.e., the CPU core and the DMA controller. In this way, we can think of a very responsive system that performs some small operations in a fast, strict, real-time environment, made possible with the internal programmable logic, and on the other hand, the high-level acquisition layer that is deployed within a complete operative system running on the processing system side.

The RedPitaya legacy software bundle (Red Pitaya OS 1.04-7) comes with several already-coded components such as a quite-fast oscilloscope, a frequency spectrum analyzer and some other useful tools. All of them are implemented using high-level software-defined components that, in turn, rely on one single specific FPGA firmware implementation that runs underneath. Although all these applications seem very promising, they effectively lack a comprehensive recording system fitting the actual performance that both the ADC and the Zynq could provide. The problem resides in the firmware implementation that is suited to record the input ADC data to an internal circular buffer that is eventually accessed by the oscilloscope application to plot the curve. To achieve a reliable complete recording of the overall input signal, the firmware had to be modified, enabling a well-fitted buffer handling between the internal logic part and the processing part. More specifically a complete recording pipeline of this kind that exploits a FIFO buffer and a DMA transaction to deploy acquired chunks of data directly into the system memory was implemented and is described in [31].

The complete handling of acquired data passes through the described internal high bandwidth communication that is possible thanks to the fast interconnection between the two subsystems (PL and PS) residing in the same chip. Besides this effective internal transaction of the acquired data from the ADC to the system memory, a specific Linux system kernel module handles all the custom parametrization of the acquisition system. Many different acquisition modes were implemented and can be then selected: a so-called “streaming” acquisition that is meant to acquire continuously from a slowly changing input signal, and a “triggered” acquisition where we adhere to the typical transient recorder feature of a classical fast DAQ device.

In both cases, the output complies with the MDSplus mdsip segmented protocol. Indeed, the overall SPIDER-CODAS acquisition system relies on the MDSplus framework to orchestrate the acquisition of the many diagnostics involved. In particular, the segmented acquisition system is a data stream that flows from the device to the experiment storage system scattered by chunks called segments. The idea of the segmented acquisition is that a segment over the data payload also has an idea of the acquisition time that those data relate to, so all the segments can be identified both from the sequential entry into the storage system but also from their time boundaries.

In the streaming implementation, all the segments are sent to the central CODAS at regular intervals to make the system able to refresh the data visualization plots with the newly acquired points. This makes it possible to see the experiment signals with a flow of plot updates and to track the recording at each instant.

On the other hand, there are situations where we want to record signals where all the information is contained in a very rapid time slot where the signal varies with a high-frequency spectrum, and then the information goes down all over long periods of time. In such a situation, the most compelling solution is the transient recording where either an internal or an external trigger is able to activate fast data acquisition that lasts in the internal memory, and that is then spooled to the central acquisition out of sync. If the internal triggering system is active, the recorder acts as a standard oscilloscope and the device records upon a particular change in the input signal; contrariwise, the external trigger can be set to fire the acquisition from both an electrical timing event (timing highway) or a network UDP multicast packet (MDSplus event).

So, depending on the nature of the input signal and the kind of sensor applied, the acquisition mode was also chosen. The DC sensor data were continuously acquired with a typical rate of 10 Ksps (CONTINUOUS acquisition mode), while the AC components acquired by the CTs were recorded either with fixed time frames during the pulse blips or with complete asynchronous MDSplus events that came from the synchronization of the experiment phases in CODAS (i.e., the start of the extraction grid power supply and others). In both cases, the acquisition modes are named SLOW or FAST, with typical sampling frequencies of 300 Ksps and 10–25 Msps, respectively. Alternatively, the AC sensors can be acquired at low frequency for the entire pulse using the CONTINUOUS mode if required.

### 5.5. Calibration

**Figure 21:**
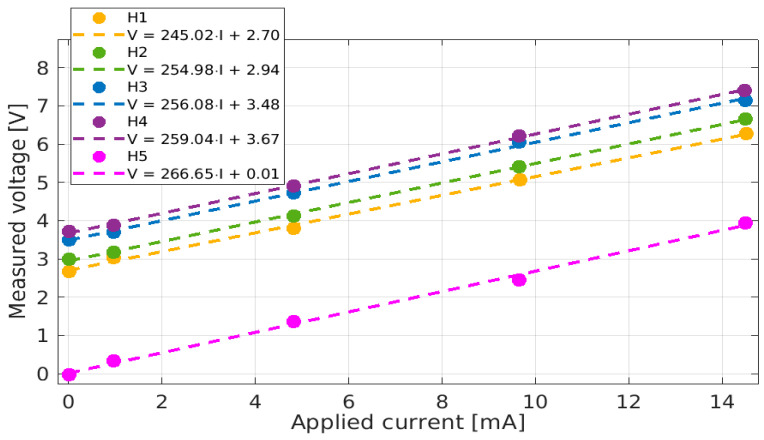
Calibration in SPIDER of the DC component of the DC sensors.

After the installation of the BCM in the vessel, a thorough calibration of each of the sensors was performed before closing the vacuum vessel. This was conducted by inserting a cable through the beam apertures of the sensor groups and applying various signals using a remote-controlled Red Pitaya function generator. A remote Red Pitaya oscilloscope monitored the sensors’ responses, as well as the signals sent through the aperture, whose precise current values were determined using a shunt resistor. Figure 21 shows the calibration of the five DC sensors: although the offset values varied significantly from one sensor to another, the sensitivities were reasonably close. The gains calculated from the calibration ranged from 245 mV/mA to 266.6 mV/mA, keeping within 1% of the value of 250 mV/mA targeted by the circuit design.

**Figure 22:**
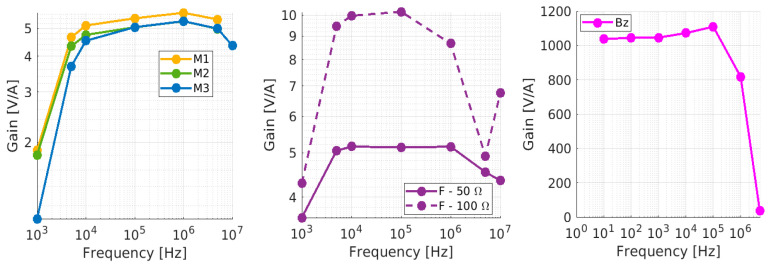
Calibration in SPIDER of the current transformers.

The frequency response of the AC sensors is shown in Figure 22. All of the sensors present a quasi-constant transfer function in the region 10 kHz–1 MHz, with sensitivities exceeding 5 mV/mA. The Bergoz offers remarkable sensitivity, 1000 mV/mA for a bandwidth of 10 Hz to 1 MHz (−3 dB). Finally, the custom transformer’s frequency response highlights the importance of the choice of the parallel resistor: opting for 50 Ω instead of 100 Ω decreases the maximum sensitivity by a factor of two but helps to flatten the response in the central region.

## 6. Experimental Results

The BCM diagnostic was partially operational during the SPIDER experimental campaigns S19 and S20 (December 2020 to March 2021—beam characterization without cesium) and fully operational during S21 (April 2021 to July 2021—cesium operation).

### 6.1. DC Results

**Figure 23:**
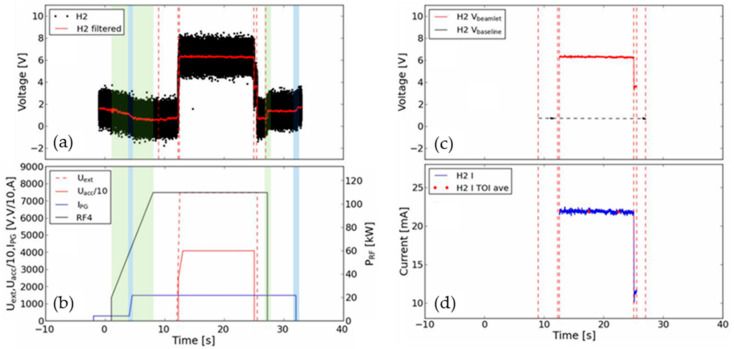
The first 40 s of shot 9145 for sensor H2 (with cesium in the SPIDER source): (**a**) sensor voltage signal (black) and 500 pt. average (red); (**b**) extraction, acceleration, filter field current and RF power signals (for the RF generator closest to the sensor); (**c**) averaged sensor voltage during beam extraction (red) and during plasma steady state for baseline (black); (**d**) beamlet current calculated from sensor voltage (blue) and ±1 s average at programmed times of interest (TOIs). The red dotted lines represent changes in power supply parameters shown in (**b**).

Figure 23 shows an example of the acquired voltage signal from one of the DC sensors during a SPIDER beam extraction pulse with cesium in the source, and the subsequent analysis required to calculate the beamlet current. The raw BCM signal (black points) and a 500-point moving average (red line) are given in Figure 23a. The sensor has a non-zero voltage offset that varies from sensor to sensor as described in previous sections. Due to the magnetic nature of the DC sensor, the voltage also has a dependency on the plasma and filter stray field as well as the current of the H^−^ passing through it. Figure 23b shows the reference settings for the RF power, plasma grid current and extraction and acceleration voltages. As the RF power or plasma grid current changes (green and blue highlights in Figure 23a,b) the sensor voltage changes with it. To ensure a steady voltage baseline before and after beam extraction, at least two seconds of plasma with stable RF power and plasma grid current are required (time between the first and last pair of red dashed lines in Figure 23a). The increase in sensor voltage during the application of extraction and acceleration voltages is therefore solely attributed to the beam passing through the sensor. The beamlet current $I_{beamlet}$ (blue in Figure 23d) is given by (6):

(6) Ibeamlet=Vbeamlet−VbaselineGs

where G_s_ is the individual sensor gain from the previous section. The voltage in the beam extraction $V_{beamlet}$ and the baseline voltage in the steady-state plasma $V_{baseline}$ are shown in red and black in Figure 23c, respectively.

The noise on the raw BCM signal at ±2 V is quite large, which, with a gain of 250 V/A, corresponds to ±4 mA. In the case of surface H^−^ production with cesium, the beamlet currents of around 20 mA result in a clear increase in sensor voltage, as in Figure 23. In volume production, with lower beamlet currents, the change in the sensor voltage with beam extraction is less clear. However, with signal averaging, the beamlet current can still be measured successfully [32].

**Figure 24:**
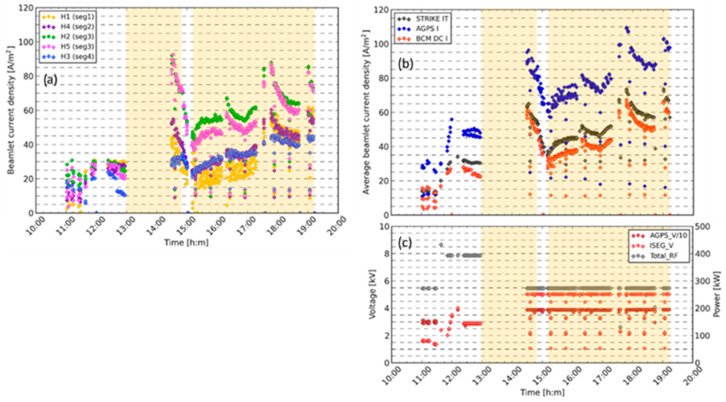
BCM DC results from the first day of SPIDER cesium operation: (**a**) beamlet current density for the five DC sensors; (**b**) average beamlet current density for the BCM, STRIKE electrical and AGPS electrical measurements; (**c**) total RF power and extraction and acceleration voltages. The times when the cesium ovens were open are highlighted in orange.

The diagnostic was used extensively during the SPIDER cesium campaign. Figure 24 shows the first day of cesium operation, with an approximately threefold increase in current density after the introduction of cesium, even with a lower total RF power. There is an observed inhomogeneity in the beamlet currents (Figure 24a). This is due to the differing availability of H^−^ at the point of extraction. There are several factors affecting the availability of H: magnetic drifts causing vertical asymmetry in the plasma density, inhomogeneous cesium deposition on the plasma grid and the position of the beamlet within the respective beamlet group (H2 and H4 in the beamlet group core and H1, H2 and H5 at the group edge) [33].

The average beamlet current measured by the BCM compares favorably with the STRIKE electrical measurement (Figure 24b). With the current PG mask STRIKE measures, the electrical current, due to 28 beamlets, spread over 9 out of 16 tiles, while the BCM measures 5 individual beamlets. The STRIKE electrical measurement requires a positive bias of up to 100 V to collect electrons emitted from the surface due to beam particle impacts. This bias can also collect electrons created in the vessel due to beam–gas interactions, which increases the measured current above the beam current alone [34].

### 6.2. AC Results

For the AC sensors, the different acquisition modes (CONTINUOUS, SLOW and FAST) were all tested and provided a number of interesting results. When operating in the continuous acquisition configuration, the sensors (in particular, the Bz sensor, which has high sensitivity at low frequencies) are capable of measuring the sudden current rise at the beginning and end of extraction and acceleration. This can serve as an additional measurement of the DC at beam startup.

**Figure 25:**
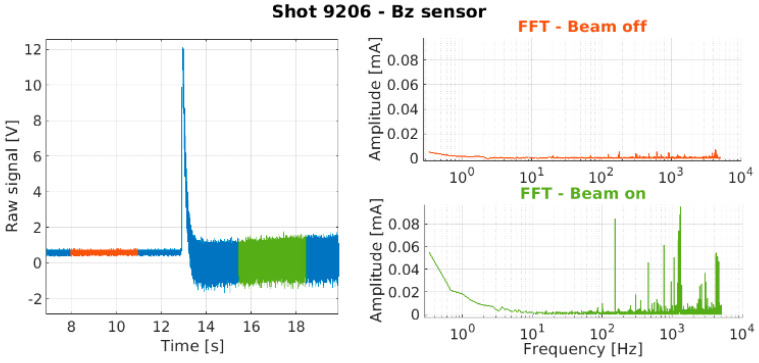
Bz measurements before extraction (orange) and during extraction (green). The FFT related to the same time intervals are reported with the same colors (right figure).

The continuous configuration is also useful as a tool to compare the measurements with and without beams. A clear difference can be observed when performing fast Fourier transforms (FFT) on the signals before or during extraction, the latter displaying a large number of peaks at amplitudes much higher than the former (Figure 25), therefore confirming that the frequencies revealed by the measurements are indeed present in the beam and not artifacts of the ambient noise. The maximum frequency shown on these particular FFTs is 5 kHz, i.e., half of the sampling frequency, as dictated by the Nyquist–Shannon sampling theorem.

**Figure 26:**
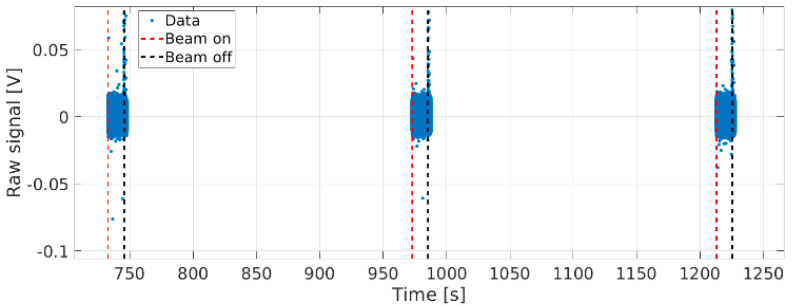
M1 measurement in SLOW acquisition mode—shot 9205.

**Figure 27:**
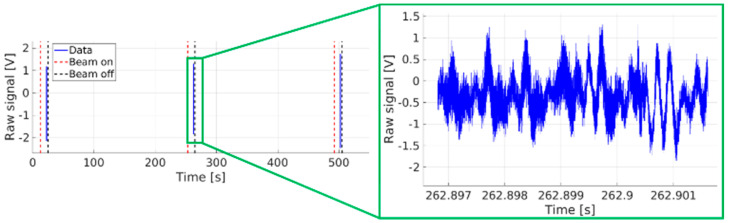
Bz measurement in FAST acquisition mode. Several frequencies can be discerned from the raw signal by the naked eye (e.g., at ∼4 kHz in this case)—shot 9342.

When operating in the SLOW or FAST acquisition modes, the sensors measure the data only during beam extraction. The sampling frequency is much larger than during continuous acquisition and allows for studies at much higher frequencies, provided that the measurement time window is reasonably short (Figure 26 and Figure 27).

**Figure 28:**
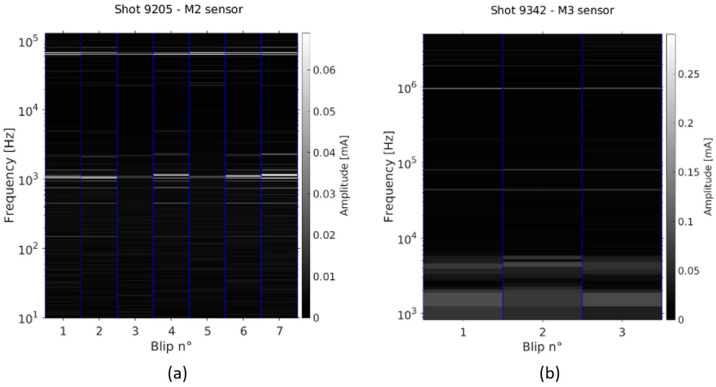
Examples of: (**a**) SLOW and; (**b**) FAST acquisition spectrograms.

When examining the FFTs performed on the AC signals, monitoring the changes in the frequency and amplitude of the outstanding peaks from one blip to another is not a trivial task to perform with the naked eye. This exercise can be facilitated by producing spectrograms, where the FFTs are plotted side by side, using the y-axis to annotate the frequencies and color-coding to represent the amplitude of the peaks (Figure 28). The important peaks identified in the FFTs are easily recognized and can be correlated with the changes in the source parameters from one blip to another. Comparing the spectrograms of several sensors can also help to understand the similarities or differences between the individual beamlets. An example of a spectrogram of measurements using the SLOW acquisition mode is shown in Figure 28a, where peaks in the range of 50–100 kHz and others of the order of the kHz stand out. The RF generator frequencies (∼1 MHz) are clearly visible on the spectrogram of measurements in the FAST acquisition mode (Figure 28b).

The BCMs were used to perform an in-depth characterization of the AC component of the SPIDER beam during the first campaign with cesium evaporation. Recurring oscillations were found, their amplitudes were measured and compared to the DC values of the beamlet currents and the frequencies were correlated with those of the RF oscillators and other power supplies when possible [35].

## 7. Conclusions

A non-intercepting system aimed at measuring the current of individual beamlets of the SPIDER experiment across the extracting area was designed, installed and widely exploited during the SPIDER S20 (March 2021) and S21 (April–November 2021) experimental campaigns, referring to negative ion production by volume and surface production (cesium injection) mechanisms, respectively.

The design of this system was undertaken within a reduced time window due to the scheduled SPIDER shutdowns, representing the only possibility for the installation of this diagnostic inside a vacuum vessel since the operation with a reduced number of beamlets was foreseen only during these campaigns. For this reason, the available off-the-shelf instruments were considered and developed against the SPIDER operational requirements, and a custom wideband current transformer based on nanocrystal materials was developed in parallel with the procurement of the former. All the instruments were extensively tested before their installation in SPIDER to assess their behavior in a vacuum, considering heat management, outgassing, transfer function and overall behavior. After having identified a temperature threshold below which the instruments work properly, thermal simulations were also carried out to assess the maximum working temperature of the instruments inside SPIDER due to thermal radiation to verify if it was compatible with the proper operation of the sensor.

The BCM system was also designed considering the overvoltage of the grounded grid (where the sensors should have been fastened) expected during breakdowns; in particular, strategies based on sensor insulation, surge arresters, insulated power supplies (PSSs) and data acquisition systems (DASs) were adopted to assure adequate reliability. Furthermore, a cheap and modular DAS and PSS, whose components were easily procurable and replaceable, was designed.

The DAS design was based on six Red Pitaya boards, whose legacy software was completely rewritten from an in-house version capable of recording and storing the measurements on the SPIDER database in real time and synchronizing them with all of the other pulse parameters and diagnostics, with a trigger provided by the CODAS system. Furthermore, the implementation of several pre-defined features (sample rate, record length and delay time from trigger signal) provided a very flexible DAS.

This system provided measurements of the current of single beamlets for the first time within a bandwidth from DC up to more than 10 MHz. The first feature permitted the assessment of beam uniformity as a function of the plasma parameters. The AC bandwidth allowed this identification and thus confirmed the hypothesis that the beam current oscillates both around the RF fundamental frequency (1 MHz) as well as at the lower beating frequencies (kHz range) among the generators.

Dedicated data analyses exploiting the large amount of data collected by the BCM system during all the foreseen campaigns are in progress with the purpose of characterizing the beam features resolved in both time and space with respect to all the relevant pulse parameters.

## Figures and Tables

**Table 1:** Identified main requirements for the BCM diagnostics.

Requirements	Value	Note
Current full-scale	≥40 mA	Max beamlet current I_b_ for the nominal accelerated D-current of 50 A
Current resolution	≤1 mA	Max allowed beam non-uniformity for I_b_ = 10 mA (early phase of surface production operation)
Sensitivity	≥5 mV/mA	Typical ADC sensitivity considering noise
Bandwidth	DC-10 MHz	Beatings in the kHz range and MHz harmonics due to RF
Clearance	≥20 mm	Beamlet cross-section at GG, divergence and deflection
External diameter	100 mm	Minimum distance between adjacent beamlets, divergence
Repeller voltage	≥100 V	STRIKE potential

**Table 2:** CT parameters for model and measurement.

CT Parameter	Value
r_i_ [m]	0.03
r_o_ [m]	0.045
h [m]	0.02
N	10
μ_r_	50,000
R_s_ [Ω]	0.22
R_c_ [Ω/m]	0.048
L_c_ [nH/m]	250
C_c_ [pF/m]	100
L_c_ [m]	15
R_l_ [Ω]	50
R_o_ [MΩ]	1
C_o_ [pF]	50
K [V/A]	4.9
f_low_ (−3 dB) [Hz]	1000

**Figure A1:**
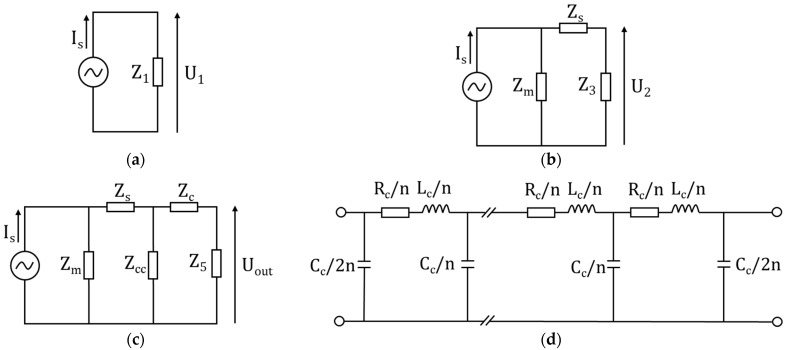
CT equivalent circuit using grouped impedances: (**a**) starting with the total impedance; (**b**,**c**) splitting the impedances by treating them as voltage dividers; (**d**) n π-junction transmission line.

## Data Availability

Not applicable.

## Appendix A

The equation for the transfer function K of the current transformer circuit given in Figure 3b can be calculated by treating the circuit as a series of voltage dividers (Figure A1). Grouping the impedance of the entire circuit as $Z_{1}$ (Figure A1a), the voltage across the circuit $U_{1}$ is given by:

(A1) $$U_{1}=I_{s}Z_{1}$$

$U_{1}$ is also the voltage drop across the magnetizing impedance $Z_{m}$ and is equal to the sum of the voltages across $Z_{s}$ and $Z_{3}$. Treating the circuit in Figure A1b as a voltage divider, the voltage across $Z_{3}$, $U_{2}$, can be expressed as:

(A2) $$U_{3}=U_{1}\frac{Z_{3}}{Z_{3}+Z_{s}}$$

In a similar manner the voltage across $Z_{5}$ (Figure A1c), which is the measured output voltage $U_{out}$, is:

(A3) $$U_{out}=U_{3}\frac{Z_{5}}{Z_{5}+Z_{c}}$$

The grouped impedances $Z_{1}$, $Z_{2}$, $Z_{3}$, $Z_{4}$ and $Z_{5}$ used in the MATLAB model are calculated from Figure 3c using the series and parallel impedance combinations:

(A4) $$Z_{5}={\left(\frac{1}{Z_{cc}}+\frac{1}{Z_{l}}+\frac{1}{Z_{o}}\right)}^{-1}$$

(A5) $$Z_{4}=Z_{c}+Z_{5}$$

(A6) $$Z_{3}={\left(\frac{1}{Z_{cc}}+\frac{1}{Z_{4}}\right)}^{-1}$$

(A7) $$Z_{2}=Z_{s}+Z_{3}$$

Substituting (A1), (A2), (A4)–(A7) and (1) into (A3) gives the transfer function from (4):

(A8) $$K=\frac{1}{N}.\frac{Z_{1}Z_{3}Z_{5}}{Z_{2}Z_{4}}$$

The individual impedances from Figure 3b used in the model are:

(A9) $$Z_{o}={\left(\frac{1}{R_{o}}+j\omega C_{o}\right)}^{-1}$$

(A10) $$Z_{l}=R_{l}$$

(A11) $$Z_{cc}=\frac{2}{{j\omega C}_{c}}$$

(A12) $$Z_{c}=R_{c}+j\omega L_{C}$$

(A13) $$Z_{s}=R_{s}$$

(A14) $$Z_{m}={\left(\frac{1}{R_{m}}+\frac{1}{j\omega L_{m}}\right)}^{-1}$$

The above formulas are valid when the transmission line is treated as a single π-junction. When $n_{c}$ π-junctions are used in the model (Figure A1d), the transfer function becomes:

(A15) $$K=\frac{1}{N}.\frac{{Z_{1}}^{’}{Z_{3}}^{’}{Z_{5}}^{’}}{{Z_{2}}^{’}{Z_{4}}^{’}}$$

where:

(A16) $${Z_{5}}^{’}={\left(\frac{1}{{n_{c}Z}_{cc}}+\frac{1}{Z_{l}}+\frac{1}{Z_{o}}\right)}^{-1}$$

Calculating ${Z_{4}}^{’}$ and ${Z_{3}}^{’}$ becomes an iterative process over n steps from 1 to $n_{c}$. For n=1:

(A17) $$Z_{4n}=\frac{Z_{c}}{n_{c}}+{Z_{5}}^{’}$$

(A18) $$Z_{3n}={\left(\frac{2}{{n_{c}Z}_{cc}}+\frac{1}{Z_{4n}}\right)}^{-1}$$

For $n=2\to n_{c}-1$:

(A19) $$Z_{4n}=\frac{Z_{c}}{n_{c}}+Z_{3n-1}$$

(A20) $$Z_{3n}={\left(\frac{2}{{n_{c}Z}_{cc}}+\frac{1}{Z_{4n}}\right)}^{-1}$$

For $n=n_{c}$:

(A21) $$Z_{4n}=\frac{Z_{c}}{n_{c}}+Z_{3n-1}$$

(A22) $$Z_{3n}={\left(\frac{1}{{n_{c}Z}_{cc}}+\frac{1}{Z_{4n}}\right)}^{-1}$$

Then:

(A23) $${Z_{4}}^{’}=\prod_{1}^{n_{c}}Z_{4n}$$

(A24) $${Z_{3}}^{’}=\prod_{1}^{n_{c}}Z_{3n}$$

(A25) $${Z_{2}}^{’}=Z_{s}+{Z_{3}}^{’}$$

(A26) $${Z_{1}}^{’}={\left(\frac{1}{Z_{m}}+\frac{1}{{Z_{2}}^{’}}\right)}^{-1}$$
